# Real-world experience with the new pulsed solid-state Thulium: YAG laser (Thulio) for endoscopic enucleation of the prostate

**DOI:** 10.1007/s00345-024-05141-8

**Published:** 2024-08-02

**Authors:** Maximilian Ferry von Bargen, M. Glienke, S. Tonyali, A. Sigle, K. Wilhelm, M. Schoenthaler, C. Gratzke, A. Miernik

**Affiliations:** 1 Department of Urology, Faculty of Medicine, University of Freiburg - Medical Centre, Hugstetter Str. 55, 79106 Freiburg, Germany; 2 Department of Urology, Istanbul University Istanbul School of Medicine, Istanbul, Turkey

**Keywords:** Benign prostate hyperplasia (BPH), Endoscopic enucleation of the prostate (EEP), Holmium laser enucleation of the prostate (HoLEP), Thulium laser enucleation of the prostate (ThuLEP)

## Abstract

**Purpose:**

The solid-state Thulium laser (Tm: YAG) is a novel alternative to the widely used Holmium laser for endoscopic enucleation of the prostate (EEP) due to its relatively high peak power. The aim of this study was to examine the efficacy and safety of a new pulsed Tm: YAG laser in its first application in humans.

**Methods:**

Data were retrospectively collected for the first 103 patients who underwent EEP with a new pulsed solid-state Tm: YAG laser (Thulio^®^, Dornier MedTech Systems GmbH, Weßling, Germany). Peri- and postoperative data were assessed. Procedure-specific complications were graded using Clavien-Dindo Classifications (CDC). Patients were interviewed 15 months after the surgery to evaluate functional and long-term outcomes. Statistical analysis was performed with Statistical Package for the Social Sciences (SPSS^®^).

**Results:**

The mean preoperative prostate volume was 105.6 ± 55.0 ml. Median enucleation speed was 4.1 g per minute (range 1.1–9.7). Short-term postoperative complications occurred in 21 patients (20.4%), but no high-grade complications (CDC ≥ IV) were observed. Five patients suffered gross haematuria and required reintervention (CDC IIIb; 4.9%). After 15 months, 76 patients (73.8%) participated in the follow-up interview, where seven patients (9.2%) reported complications, including two reinterventions for urethral strictures (CDC IIIb; 2.6%). Most patients reported an improvement in continence (54.0%) and urine stream (93.4%), but no difference in erectile function (81.6%). No persistent dysuria was reported. Patient satisfaction with the surgery results was very high (96.1%).

**Conclusion:**

Endoscopic enucleation of the prostate with the new pulsed solid-state Tm: YAG laser is a safe and effective option for surgical BPH treatment.

**Trial registration:**

German Clinical Trials Register number: DRKS00031676. Registration date: 10 May 2023, retrospectively registered.

## Introduction

Lower urinary tract symptoms (LUTS) due to benign prostate hyperplasia (BPH) have long been predominantly treated with simple prostatectomy and transurethral resection of the prostate (TURP). Over the past 20 years, several laser-based procedures have been introduced as treatment alternatives in clinical practice. Laser endoscopic enucleation of the prostate (EEP) was shown to be more effective and caused fewer complications and less blood loss than TURP and open prostatectomy [1], but was also found to have a relatively steep learning curve of up to 60 cases [2, 3].

Holmium laser enucleation of the prostate (HoLEP) was first described in 1998 by Fraundorfer & Gilling [4]. It is now the gold standard surgical treatment for LUTS attributable to BPH and first-line treatment for prostatic glands exceeding 80 cm³ in both the European Association of Urology and American Urology Association guidelines [5, 6]. HoLEP has been proven to be safe and efficient with excellent long-term clinical results [7]. It uses a pulsed Holmium: yttrium-aluminium-garnet laser (Ho: YAG) with a 2120 nm wavelength and a peak power of up to 10 kW. The laser energy rapidly heats the water medium, causing a cavitation bubble to form which emits a shock wave exerting local pressure [8]. This facilitates dissecting the prostate adenoma from the prostate’s pseudo-capsule, thus enabling anatomic prostate enucleation [8]. Although the heat from the steam bubble coagulates the wound and stops superficial bleeding, this coagulation effect is considered irregular and suboptimal [8]. An attempt to reduce this photodynamic effect is the new low peak power Ho: YAG laser [9].

Two main Thulium laser types are used for EEP as alternatives to Ho: YAG lasers: the continuous wave Thulium: YAG (Tm: YAG) laser used for enucleation with Tm: YAG support (ThuLEP), and the Thulium fiber laser (TFL).

The TFL employs an optical fibre with a central Tm-doped core as its active medium [10]. Concerning tissue cutting they might be more precise than the Ho: YAG laser due to their lower peak power up to 500 W producing four-fold smaller vapour bubbles and ten-fold lower local pressure [11]. Both continuous and pulsed-wave TFLs were reported as equally safe and effective for laser enucleation of the prostate [12].

Continuous wave Tm: YAG is a solid-state laser with Tm-doped YAG crystals as active medium and resonator to create the laser beam with a continuous wave [10]. It might be used as an alternative to TFL for EEP due to its superior ablative and coagulative properties [10], and may be superior to other lasers like Ho: YAG thanks to minimal blood loss, high ablation rate causing minimal thermal injury, and precise incision [13]. A systematic review and meta-analysis found continuous wave ThuLEP resulted in greater intraoperative safety and faster alleviation of symptoms than HoLEP [14]. Another systematic review and meta-analysis reported minor advantages regarding blood loss and transient incontinence for continuous wave ThuLEP over HoLEP [15]. However, the continuous wave Tm: YAG laser does not permit the employment of cavitation bubble explosions for tissue dissection [16].

To overcome the shortcomings of existing lasers, pulsed solid-state Tm: YAG lasers were introduced [17, 18]. They also use a Tm-doped YAG crystal as energy source, but can be employed in a pulsed operating mode [19]. The RevoLix HTL (OmniGuide, Cambridge, MA, USA) can be operated in both continuous wave and pulsed mode up to 300 Hz. In an ex vivo model, the incision depth and laser damage was easier to control with pulsed Tm: YAG compared to continuous wave Tm: YAG and pulsed Ho: YAG lasers [17]. However, its peak power of 1,300 W is relatively low.

A novel pulsed solid-state Tm: YAG laser with a much higher peak power of 3,700 W was recently introduced. A higher peak power results in a larger cavitation bubble, which facilitates prostate enucleation and improves stone disintegration, particularly in hard stones [20]. So far, there have been few experimental studies assessing its performance [18, 19]. To the best of our knowledge, no clinical study reporting laser EEP outcomes has been published. We describe the very first experience using this novel pulsed solid-state Tm: YAG laser for EEP due to BPH in human.

## Materials and methods

This study was performed in line with the principles of the Declaration of Helsinki. Approval was granted by the local ethics committee of the Albert-Ludwigs-University of Freiburg, Germany (Approval number: 22-1465-S1-retro). We retrospectively analyzed the medical records of all consecutive patients who underwent laser EEP supported by the novel pulsed solid-state Tm: YAG laser in our institution between May and December 2022. All included patients had been diagnosed with LUTS due to BPH who had failed to respond to medical treatment and were with or without refractory urinary retention and/or refractory haematuria attributable to prostate enlargement.

Demographic and clinical data were extracted by reviewing the patients’ electronic medical records. Baseline characteristics included age, prostate volume (measured by transabdominal ultrasound), prostate specific antigen (PSA), International Prostate Symptom Score (IPSS) and quality of life (QoL) questionnaires, uroflowmetry, post-void residual (PVR) bladder scan, and ASA (American Society of Anesthesiologists) risk classification.

Antiplatelet therapy with acetylsalicylic acid was not stopped preoperatively. Anticoagulation with vitamin K antagonists was bridged while direct oral anticoagulants were paused two days before the procedure.

In addition, hospital visits at our department that were related to the procedure within one month after the surgery were extracted from individual patient records.

The severity of procedure-specific complications was scored according to the Clavien-Dindo Classification (CDC) system. We documented short and long-term complications. Procedure-specific complications were defined as symptomatic urinary tract infection, bladder perforation, urinary retention, and reintervention for haematuria [21]. Peak pain after surgery was measured via the numeric rating scale. A telephone follow-up interview was carried out 15 months after the surgery. Here, the patients were asked 10 questions about complications, continence, LUTS, erection, and overall satisfaction with the surgery.

### Surgical procedure

The novel laser is a diode pumped pulsed solid-state Tm: YAG laser (Thulio, Dornier MedTech Systems GmbH, Wessling, Germany) which emits laser radiation at a wavelength of 2013 nm with a 100 W maximum average power and a peak power up to 3,700 W. A 600 μm laser fibre and 26 French continuous flow resectoscope (Karl Storz SE & Co. KG, Tuttlingen, Germany) were used for the procedures with the laser power settings at 100 W at 2 J and 50 Hz based on previous experience [18]. The pulse duration was adjusted to balance effective tissue interaction with safety, tailored to the Tm: YAG laser’s unique characteristics. All EEP procedures were carried out by three experienced surgeons (having done more than 250 EEPs) and applying the same en-bloc technique (three horse shoe-like incision) [22]. Following our hospital protocol for EEP, a high frequency current cautery device (Erbe Elektromedizin GmbH, Tübingen, Germany) with a monopolar roller ball probe (Richard Wolf GmbH, Knittlingen, Germany) was used as an additional haemostatic measure after every enucleation. Standard saline (0.9%) solution was used to irrigate, and the bags were hung 60 cm above the operating table. Tissue morcellation was performed with the Piranha device (Richard Wolf GmbH, Knittlingen, Germany). Following morcellation of the prostate tissue, a standard 20 Fr three-way catheter was inserted with continuous bladder irrigation, respectively.

### Statistical analyses

Data were collected and statistics analyzed using Statistical Package for the Social Sciences (SPSS^®^) version 26.0 (Chicago, IL, USA). The Kolmogorov-Smirnov test was used to test if our data had normal distribution. Normally distributed data were determined according to the mean and standard deviation, whereas non-normally distributed data were reported as median and range. The Fischer’s exact test was used to determine statistical significance. A p-value < 0.05 was considered statistically significant.

## Results

A total of 103 patients were included in this study. The population characteristics as well as peri- and postoperative data are reported in Table 1. The patients had a mean age of 70.4 ± 7.7 years. The mean preoperative prostate volume was 105.6 ± 55.0 ml and median PSA was 5.3 ng/ml (range 0.5–132.0). The mean preoperative IPSS was 20.1 ± 6.6 with a median QoL score of 4 (range 0–5) and peak flow rate of 11.5 ± 4.9 ml/sec. The median preoperative PVR volume was 85 ml (range 0-1300). The median enucleated prostate volume was 65 g (range 16–361). Surgeries took a median 38 min (range 16–182) to complete, with 17 min (range 8–72) to perform enucleation and 7 min (range 2–69) to perform morcellation. The enucleation speed was 4.1 g/min (range 1.1–9.7) and the laser energy was 629.0 J per g of enucleated prostate (range 248.9-2,018.8). After surgery, catheters stayed in place for a median 2 days (range 2–5), and patients were discharged from hospital after a median stay of 4 days (range 2–8).

**Table 1:** Preoperative population characteristics

	*N*	%	Mean	SD	Median	Range
Preoperative descriptive data						
Age (years)	103		70.4	7.7	71	54–88
Prostate volume (ml)	102		105.6	55.0	98.0	48–500
PSA (ng/ml)	101		7.9	13.9	5.3	0.5–132.0
IPSS	59		20.1	6.6	20.0	6–33
QoL	52		3.5	1.2	4.0	0–5
PFR (ml/s)	61		11.5	4.9	12.1	3.0-24.6
Preoperative PVR (ml)	80		147.2	184.3	85.0	0–1,300
Hb (g/dl)	103		14.5	1.3	14.5	8.3–16.5
Antiplatelet therapy	18	17.5				
Anticoagulant therapy	6	5.8				
ASA risk classification	103		2.28			1–3
Peri- and post-operative descriptive data						
Prostate volume (g)	103		76.6	46.4	65	16–361
Duration of surgery (min)	102		40.8	21.7	38	16–182
Duration of enucleation (min)	102		18.3	8.9	17	8–72
Duration of morcellation (min)	102		10.2	10.1	7	2–69
Total energy (J)	102		49,572	27,138	44,536	13,690 − 248,000
Energy per gram prostate (J/g)	102		750.5	375.4	629.0	248.91-2,018.75
Speed of enucleation (g/min)	102		4.4	1.9	4.1	1.1–9.7
PVR (ml)	101		32.2	34.8	25	0-190
Peak pain after surgery (NRS)	103		1.4	1.5	1	0–8
Duration of catheterization (days)	102		2.2	0.5	2	2–5
Duration of hospitalization (days)	103		4.2	0.8	4	2–8
IPC	7	6.8%				

ASA: American Society of Anesthesiologists; IPC: incidental prostate cancer; IPSS: International prostate symptom score; NRS: numeric rating scale; PFR: peak flow rate; PSA: prostate specific antigen; PVR: post-void residual volume; QoL: quality of life; SD: Standard deviation

Short-term postoperative complications occurred in 21 patients (20.4%) (Table 2) and ranged from CDC I to IIIb. Four patients (3.9%) experienced a CDC II complication (postoperative fever ≥ 38 °C). Eleven patients (10.7%) developed acute urinary retention after catheter removal (CDC IIIa) and needed a recatherization. Five patients (4.9%) suffered gross haematuria necessitating a reintervention for transurethral coagulation to control bleeding (CDC IIIb); one of those patients (1.0%) needed a blood transfusion during re-surgery for bleeding, the others were managed with prolonged bladder washout without re-surgery. No correlation was observed between postoperative bleeding and the use of anticoagulants or antiplatelet drugs (not shown). No complications with a CDC higher than IIIb were observed.

**Table 2:** Complications during postoperative period (short-term) and within 15 months of surgery

		*N* (%)	*N* (%)
Clavien-Dindo Classification (CDC)		postoperative (*N* = 103)	within 15 months after discharge from hospital (*N* = 76)
	0	82 (79.6)	0
	I	1 (1.0)	0
	II	4 (3.9)	5 (6.6)
	IIIa	11 (10.7)	0
	IIIb	5 (4.9)	2 (2.6)
Type of complication			
	Fever (CDC II)	4 (3.9)	5 (6.6)
	Acute urinary retention (CDC IIIa)	11 (10.7)	0
	Reintervention for bleeding (CDC IIIb)	5 (4.9)	0
	Transfusion	1 (0.97)	0
	Reintervention for urethral stricture (CDC IIIb)	0	2 (2.6)

CDC: Clavien-Dindo classification

At 15 months after the surgery, we were able to reach 76 patients (73.1%) for the phone interview, all of whom agreed to answer the follow-up questionnaire. One patient died due to an unrelated reason (pulmonary embolism in the context of a covid infection) and 26 patients could not be reached despite repeated attempts. During this follow-up period, seven patients had a long-term complication: two patients (2.6%) required reintervention for urethral stricture (CDC IIIb; urethrotomy and transurethral bladder neck incision), and five more patients (6.6%) reported having a fever (CDC II).

The functional outcomes at the 15-month follow-up interview are shown in Table 3. Most interviewed patients (*n* = 73, 96.1%) were satisfied with the result of the surgery. An improvement in continence was observed in 41 patients (54.0%), whereas a decline in continence was reported by 6 patients (7.9%). Incontinence pads were used by 14 patients (18.4%) during the day, using a mean 1.4 (SD ± 1.0) pads/day, and 49 patients (64.5%) still had nocturia. Patients reported having to get up between 1 and 4 times per night (mean 1.4 ± 0.7). No patient suffered from dysuria after surgery and only 6 patients (7.9%) had mild urge symptoms. The urine stream of 71 patients (93.4%) improved while one patient (1.3%) experienced deterioration. Most patients reported no difference in erectile function (*n* = 62, 81.6%), whereas 6 patients (7.9%) reported an improvement and 7 patients (9.2%) described erectile function as worse than before the surgery.

**Table 3:** Functional outcomes 15 months after the surgery

*N* = 76	*N* (%)	Mean (± SD)	Compared to before surgery		
			Better, *N* (%)	No difference, *N* (%)	Worse, *N* (%)
Dysuria	0				
Incontinence pads/day	14 (18.4)	1.4 (± 1.0)			
Nocturia	49 (64.5)	1.4 (± 0.7)			
Urge	6 (7.9)		0	2 (2.6)	4 (5.3)
Continence			41 (54.0)	29 (38.2)	6 (7.9)
Erectile function			6 (7.9)	62 (81.6)	7 (9.2)
Urine stream			71 (93.4)	4 (5.3)	1 (1.3)
Overall satisfaction			73 (96.1)	0	3 (4.0)

SD: Standard deviation

Eight patients were diagnosed with prostate cancer. There was no significant difference in the complication rate of patients with or without prostate tumours regarding the functional outcome (p-value = 0.638, not shown). Furthermore, the intake of oral anticoagulation showed no significant correlation with postoperative bleeding (p-value = 0.264, not shown).

## Discussion

LUTS attributable to BPH represent one of the most common medical issues in aging men, exhibiting a prevalence that increases nearly linearly with age [23]. Roughly 80% of men above the age of 80 years suffer from LUTS [23].

In the present study, 20.4% of our patients experienced some degree of short-term complications and an additional 9.2% of the patients who completed the 15-month follow-up experienced long-term complications. All their complications were well manageable clinically and were below CDC Grade IV. Among those patients, five (4.9%) required a reintervention for bleeding control and only one (1.0%) a blood transfusion. Prostatic cancer was found to have no impact on the complication rate, and the intake of oral anticoagulation did not influence postoperative bleeding. Our complication rates resemble those reported in the literature, and confirm the safety of the newly introduced pulsed Tm: YAG laser in EEP [21]. Furthermore, the vast majority of patients (96.1%) were satisfied with the results of the surgery. These findings suggest that this technology is safe and readily applicable within a routine patient cohort.

In our real-world experience, we observed that the high-power pulsed Tm: YAG laser with a peak power up to 3,700 W facilitated correct anatomic adenoma dissection even in cases of exceptionally large prostate volumes. Despite the considerably large sizes of the prostatic glands, the average enucleation time was maintained at a relatively brief 18.3 min (with a maximum of 72 min), confirming the laser’s efficacy in EEP.

The duration of urethral catheterization in our patients might be considered long (2.2 days). However, it is important to note that although most patients were eligible for catheter removal on the same day or the day after surgery, we had to continue on catheterization over 48 h for a full case reimbursement (the currently shortest possible catheter indwelling time after ThuLEP in Germany).

This study has limitations. Our study has a single-centre design. Because of the German DRG (Diagnosis Related Groups) system affecting the duration of hospitalization and our institution’s standard operating procedures after catheter removal, patients were discharged at the third postoperative day at the earliest. Furthermore, this is a single-arm observational real-world surgical care study, so no direct comparisons to other lasers are possible. Since our data only contain information on a period of 15 months after the operation, we do not have any data on further possible long-term complications after this period. The follow-up data is based on subjective, patient-reported outcomes rather than objective evaluation methods, which were outside the scope of this retrospective study. It is further possible that the patients that could not be reached for the phone interview introduced a bias in the long-term outcomes. Additionally, the follow-up question regarding incontinence pad usage did not quantify the urine volume absorbed by the pads and included very thin “security pads” designed as a precautionary measure for minor urine leaks that may not indicate clinically significant incontinence, thereby possibly overestimating the incontinence rate. It is also possible that the complication rate in the first case series might be higher because of our relative lack of experience with the new laser system. Although the Tm: YAG laser settings used here were based on previous experience [18], no international standard exists as of yet. Finally, while the use of monopolar electrocautery is a well-established approach at our hospital, we acknowledge that it is not a standard practice and may cause an overestimation of the laser’s haemostatic properties.

## Conclusion

In the present study we demonstrated that laser EEP with the newly introduced pulsed solid-state Thulium: YAG laser is a safe and effective treatment option allowing the anatomic enucleation of a prostate, resulting in very high postoperative patient satisfaction. In future, larger and, ideally, prospective studies will be required to substantiate the genuine value and clinical efficacy of this laser system.

## References

[1] Pirola GM, Saredi G, Codas Duarte R et al (2018) Holmium laser versus thulium laser enucleation of the prostate: a matched-pair analysis from two centers. Ther Adv Urol 10(8):223–233. 10.1177/1756287218779784 30034541 10.1177/1756287218779784 PMC6048626

[2] Perri D, Pacchetti A, Morini E et al (2024) Evaluation of the learning curve for Thulium laser enucleation of the prostate in a contemporary cohort. World J Urol 42(1):246. 10.1007/s00345-024-04958-7 38643250 10.1007/s00345-024-04958-7

[3] Brunckhorst O, Ahmed K, Nehikhare O, Marra G, Challacombe B, Popert R (2015) Evaluation of the learning curve for Holmium Laser Enucleation of the prostate using multiple outcome measures. Urology 86(4):824–829. 10.1016/j.urology.2015.07.021 26254171 10.1016/j.urology.2015.07.021

[4] Fraundorfer MR, Gilling PJ (1998) Holmium:YAG laser enucleation of the prostate combined with mechanical morcellation: preliminary results. Eur Urol 33(1):69–72. 10.1159/000019535 9471043 10.1159/000019535

[5] Gravas S, Gacci M, Gratzke C et al (2023) Summary Paper on the 2023 European Association of Urology Guidelines on the management of non-neurogenic male lower urinary tract symptoms. Eur Urol 84(2):207–222 37202311 10.1016/j.eururo.2023.04.008

[6] Sandhu JS, Bixler BR, Dahm P et al (2024) Management of lower urinary tract symptoms attributed to Benign Prostatic Hyperplasia (BPH): AUA Guideline Amendment 2023. J Urol 211(1):11–19. 10.1097/JU.0000000000003698 37706750 10.1097/JU.0000000000003698

[7] Netsch C, Gross AJ (2019) Thulium laser enucleation of the prostate. Curr Opin Urol 29(3):302–303. 10.1097/MOU.0000000000000610 30950888 10.1097/MOU.0000000000000610

[8] Socarrás MR, Del Álamo JF, Sancha FG (2023) Long live holmium! Eur Urol Open Sci 48:28–30. 10.1016/j.euros.2022.07.012 36588773 10.1016/j.euros.2022.07.012 PMC9795518

[9] Bozzini G, Maltagliati M, Besana U et al (2021) Holmium laser enucleation of the prostate with virtual Basket mode: faster and better control on bleeding. BMC Urol 21(1):28. 10.1186/s12894-021-00797-5 33622326 10.1186/s12894-021-00797-5 PMC7903737

[10] Bozzini G, Berti L, Maltagliati M et al (2022) Thulium: YAG vs continuous-wave thulium fiber laser enucleation of the prostate: do potential advantages of thulium fiber lasers translate into relevant clinical differences? World J Urol. 10.1007/s00345-022-04201-1 36357602 10.1007/s00345-022-04201-1

[11] Kronenberg P, Traxer O (2019) The laser of the future: reality and expectations about the new thulium fiber laser-a systematic review. Transl Androl Urol 8(Suppl 4):S398–S417. 10.21037/tau.2019.08.01 31656746 10.21037/tau.2019.08.01 PMC6790412

[12] Perri D, Mazzoleni F, Besana U et al (2023) Pulsed-wave vs continuous-wave Thulium Fiber Laser Enucleation of the prostate (ThuFLEP): a comparison of Perioperative outcomes. Urology 178:120–124. 10.1016/j.urology.2023.05.013 37257589 10.1016/j.urology.2023.05.013

[13] Xiao K-W, Zhou L, He Q et al (2019) Enucleation of the prostate for benign prostatic hyperplasia thulium laser versus holmium laser: a systematic review and meta-analysis. Lasers Med Sci 34(4):815–826. 10.1007/s10103-018-02697-x 30604345 10.1007/s10103-018-02697-x

[14] Meng C, Peng L, Li J, Li Y, Yang J, Wu J (2022) Comparison of enucleation between thulium laser and holmium laser for benign prostatic hyperplasia: a systematic review and meta-analysis. Asian J Surg 45(2):689–697 34384678 10.1016/j.asjsur.2021.07.045

[15] Hartung FO, Kowalewski KF, von Hardenberg J et al (2022) Holmium Versus Thulium Laser Enucleation of the prostate: a systematic review and Meta-analysis of Randomized controlled trials. Eur Urol Focus 8(2):545–554. 10.1016/j.euf.2021.03.024 33840611 10.1016/j.euf.2021.03.024

[16] Ventimiglia E, Villa L, Doizi S et al (2021) Laser lithotripsy: the importance of Peak Power and Pulse Modulation. Eur Urol Focus 7(1):22–25. 10.1016/j.euf.2021.01.012 33531287 10.1016/j.euf.2021.01.012

[17] Huusmann S, Lafos M, Meyenburg I, Muschter R, Teichmann H-O, Herrmann T (2021) Tissue effects of a newly developed diode pumped pulsed Thulium:YAG laser compared to continuous wave Thulium:YAG and pulsed Holmium:YAG laser. World J Urol 39(9):3503–3508. 10.1007/s00345-021-03634-4 33728503 10.1007/s00345-021-03634-4 PMC8510916

[18] Yilmaz M, Esser J, Kraft L et al (2022) Experimental ex-vivo performance study comparing a novel, pulsed thulium solid-state laser, chopped thulium fibre laser, low and high-power holmium:YAG laser for endoscopic enucleation of the prostate. World J Urol 40(2):601–606. 10.1007/s00345-021-03825-z 34477954 10.1007/s00345-021-03825-z PMC8921029

[19] Kraft L, Yilmaz M, Petzold R, Gratzke C, Suarez-Ibarrola R, Miernik A (2022) Dusting efficiency of a Novel Pulsed Thulium:Yttrium Aluminum Garnet Laser vs a Thulium Fiber laser. J Endourol 36(2):259–265. 10.1089/end.2021.0441 34693738 10.1089/end.2021.0441

[20] Ventimiglia E, Robesti D, Bevilacqua L et al (2023) What to expect from the novel pulsed thulium:YAG laser? A systematic review of endourological applications. World J Urol 41(11):3301–3308. 10.1007/s00345-023-04580-z 37682286 10.1007/s00345-023-04580-z

[21] Capogrosso P, Fallara G, Pozzi E et al (2022) Rates and predictors of postoperative complications after Holmium laser enucleation of the prostate (HoLEP) at a high-volume center. Minerva Urol Nephrol 74(4):461–466. 10.23736/S2724-6051.21.04315-9 33887894 10.23736/S2724-6051.21.04315-9

[22] Miernik A, Schoeb DS (2019) Three horse shoe-like incision holmium laser enucleation of the prostate: first experience with a novel en bloc technique for anatomic transurethral prostatectomy. World J Urol 37(3):523–528. 10.1007/s00345-018-2418-0 30039386 10.1007/s00345-018-2418-0

[23] Madersbacher S, Sampson N, Culig Z (2019) Pathophysiology of Benign Prostatic Hyperplasia and Benign Prostatic Enlargement: a Mini-review. Gerontology 65(5):458–464. 10.1159/000496289 30943489 10.1159/000496289

